# Prognostic Value of Stromal Type IV Collagen Expression in Small Invasive Breast Cancers

**DOI:** 10.3389/fmolb.2022.904526

**Published:** 2022-05-25

**Authors:** Malin Jansson, Jessica A. Lindberg, Gunilla Rask, Johan Svensson, Ola Billing, Anoosheh Nazemroaya, Anette Berglund, Fredrik Wärnberg, Malin Sund

**Affiliations:** ^1^ Department of Surgery and Perioperative Sciences/Surgery, Umeå University, Umeå, Sweden; ^2^ Department of Medical Biosciences/Pathology, Umeå University, Umeå, Sweden; ^3^ Department of Statistics, Umeå School of Business, Economics and Statistics, Umeå University, Umeå, Sweden; ^4^ Department of Surgery, Institute of Clinical Sciences, Sahlgrenska Academy at the University of Gothenburg, Gothenburg, Sweden; ^5^ Department of Surgery, University of Helsinki and Helsinki University Hospital, Helsinki, Finland

**Keywords:** type IV collagen, breast cancer, extracellular matrix, tumor progression, tumor microenvironment

## Abstract

Breast cancer is the most common cause of cancer death among women worldwide. Localized breast cancer can be cured by surgery and adjuvant therapy, but mortality remains high for tumors that metastasize early. Type IV collagen is a basement membrane protein, and breach of this extracellular matrix structure is the first step of cancer invasion. Type IV collagen is found in the stroma of many cancers, but its role in tumor biology is unclear. Here, expression of type IV collagen in the stroma of small breast cancers was analyzed, correlated to clinically used prognostic biomarkers and patient survival. The findings were further validated in an independent gene expression data cohort. Tissue samples from 1,379 women with *in situ* and small invasive breast cancers (≤15 mm) diagnosed in 1986-2004 were included. Primary tumor tissue was collected into tissue microarrays. Type IV collagen expression in tissues was visualized using immunohistochemistry. Gene expression data was extracted from the Cancer Genome Atlas database. Out of 1,379 women, 856 had an invasive breast cancer and type IV collagen staining was available for 714 patients. In Kaplan-Meier analysis high type IV collagen expression was significantly associated (*p* = 0.026) with poorer breast cancer specific survival. There was no correlation of type IV collagen expression to clinically used prognostic biomarkers. High type IV collagen expression was clearly associated to distant metastasis (*p* = 0.002). In an external validation cohort (*n* = 1,104), high type IV collagen mRNA expression was significantly (*p* = 0.041) associated with poorer overall survival, with overexpression of type IV collagen mRNA in metastatic tissue. Stromal type IV collagen expression in the primary tumor correlates to poor breast cancer specific survival most likely due to a higher risk of developing distant metastasis. This ECM protein may function as biomarker to predict the risk of future metastatic disease in patients with breast cancers.

## 1 Introduction

Worldwide, breast cancer is the most common cancer type among women, impacting 2.2 million women each year. Most patients are successfully treated with surgery and adjuvant treatment, but for subgroups of patient mortality rates remain high. In 2020, it was estimated that 684,966 women died from breast cancer (https://gco.iarc.fr/today/home) (World Health Organization 2020). Breast cancers evolve via sequential progression thorough defined stages, starting with epithelial hyperproliferation, progressing to *in situ* and further to invasive cancer (Polyak 2007). To select the best therapy and estimate prognosis, invasive breast cancers are categorized into different molecular subtypes, including luminal A, luminal B (HER2-), luminal B (HER2+), Her2 positive and triple negative breast cancers (TNBC) (Schnitt 2010). This classification is based on gene expression profiling or surrogate grouping based on protein expression (Sorlie et al., 2003; Goldhirsch et al., 2013).

The extracellular matrix (ECM) is increasingly recognized as a major regulator of carcinogenesis and an important participant in each step of cancer progression (Place, Jin Huh, and Polyak 2011). The grouping into molecular subtypes is used to predict outcome and treatment response and is solely based on the properties of the invasive cancer cell. Studies have shown that the ECM also carries diagnostic and prognostic value, although not yet used in clinical practice (Finak et al., 2008; Bredfeldt et al., 2014). The ECM can be divided into two matrices; the interstitial matrix (IM) and the basement membrane (BM). The IM makes up the main stroma and plays a major role in cell migration, cell adhesion, angiogenesis, tissue development and repair (Theocharis et al., 2016). The BM acts as a barrier separating the epithelium from the surrounding stroma, but it also regulates cell behavior and recruitment to signaling pathways (Kalluri 2003). Type IV collagen is the major component of the BM and consists of six homologous chains, the α1-α6 chains, that assemble into three heterotrimers. The α1α2α1 heterotrimer is present in the BM of almost all tissues (Khoshnoodi, Pedchenko, and Hudson 2008), whereas the others have more restricted tissue distributions and specialized functions. In breast, the α5 and α6-chains are specifically located in BM of the mammary duct and lobule (Nakano et al., 1999). Type IV collagen is required for tissue repair processes, enables survival and function of endothelial and epithelial cells, and regulates tissue function (Theocharis et al., 2016). Degradation of type IV collagen can give rise to bioactive peptides, such as the NC1 domains of α1 (arresten), α2 (canstatin), α3 (tumstatin), α4(tetrastatin), α5 (pentastatin) and α6 (hexastatin) chains, with each of these potentially eliciting unique cellular responses (Ricard-Blum and Vallet 2019).

As the destruction of the BM is the first step in epithelial cancer invasion, type IV collagen and its fragments, have therefore been of interest in understanding tumor progression (Monboisse et al., 2014). Depending on the context, type IV collagen can have both pro- and anti-tumor effects in cancer, although the turnover and degradation of this protein is intrinsically associated with invasive cancer (Ikeda et al., 2006; Baba et al., 2008). Furthermore, it is also shown that this protein is highly expressed in the tumor stroma, and in pancreatic cancer type IV collagen acts to stimulate proliferation, migration and prevent apoptosis (Ohlund et al., 2009; Ohlund et al., 2013). In gastric cancer, high expression of the α3 chain of type IV collagen is correlated with negative prognostic factors, such as tumor size, lymphatic invasion and TNM stage (Nie et al., 2013). In colorectal cancer liver metastasis, type IV collagen is highly expressed in the stroma surrounding cancer cells indicating that this deposition may be important for metastatic growth (Nystrom et al., 2011). In breast cancer, recent studies have shown that type IV collagen or its degradation products may function as tumor markers (Mazouni et al., 2008; Lindgren et al., 2021), and that a high circulating level is associated with poor outcome in metastatic disease (Lipton et al., 2018).

## 2 Aim

The aim was to study the expression of type IV collagen in breast cancer tissue from small tumors using a large patient cohort with long follow-up to establish if expression correlates with known prognostic biomarkers and the prognosis of the patient. The results were validated in an external gene expression cohort.

## 3 Materials and Methods

### 3.1 Patient Cohort

The source population of the study was a cohort of women diagnosed with small breast cancers (defined as clinical primary tumor size of 15 mm or below), both invasive and *in situ*, in the Uppsala-Västerås region in Sweden from 1986 to 2004. There were no exclusion criteria and the total number of patients included was 1,395. Patients were monitored every 2 years through review of medical charts, and clinical data were collected with the latest follow-up done in the summer of 2019. The Regional Ethics Committee of Uppsala approved the study (record number 99 422, 2005:118, and 2005:118/2).

### 3.2 Tissue Microarray

Tissue microarrays (TMAs) were produced by collecting all primary tumor paraffin blocks. Hematoxylin and eosin sections were reviewed and areas with invasive cancer selected. Representative areas were then punched and brought into recipient paraffin blocks to construct TMAs consisting of two cores (diameter 1 mm) of each tumor. Three to 4 μM thick sections were cut from the TMAs and transferred to glass slides.

### 3.3 Immunohistochemistry

#### 3.3.1 Routine Staining of Breast Cancer Biomarkers

Routine staining was performed for hematoxylin and eosin using standard protocols. A panel of clinically used breast cancer biomarkers (nuclear grade, estrogen receptor (ER), progesterone receptor (PR), proliferation marker Ki67 and HER2 gene amplification) were assayed and scored by two subspecialized breast pathologists (GR and AN) at the Department of Pathology of Umea University Hospital according to externally validated and accepted guidelines. High Ki67 expression was defined as ≥ 10% stained cells and low Ki67 as < 10%. ER and PR were scored as positive (≥10%) and negative (<10%). Disagreement in the scoring was resolved through discussion.

#### 3.3.2 Type IV Collagen Staining

Immunohistochemical staining for type IV collagen was performed using deparaffinized sections treated with proteinase for 15 min at room temperature (RT). The slides were washed and incubated with blocking buffer consisting normal goat serum (20%) in PBS for 30 min at RT. The primary antibody used was rabbit anti-collagen IV (AB748, Millipore, Billerica, United States) diluted 1:50 in blocking buffer and the sections were incubated for 2 h in RT. The sections were then washed in PBS and incubated with the biotinylated secondary antibody, diluted 1:200 in blocking buffer, for 30 min in RT. Finally, the slides were washed in PBS followed by diaminobenzidine tetrahydrochloride (DAB) as a chromogen.

### 3.4 Scoring of Type IV Collagen Expression

The TMA consisted of two sections from each tumor, and the expression of type IV collagen in the tumor stroma was graded in a three-level scoring system where 1 = low expression, 2 = moderate expression and 3 = strong expression. The scoring was made by two independent researchers (MJ and AB) who were blinded to outcomes when assessing the samples. Both assessors had previous experience of type IV collagen expression scoring and any disagreements were resolved through discussion.

### 3.5 Surrogate Molecular Subtypes

The molecular subtype classification was made using the criteria according to the guidelines of St. Gallen international Expert Consensus (Maisonneuve et al., 2014; Ehinger et al., 2017). Nuclear grade was used instead of histologic grade since neither tubule formation nor mitotic activity can be adequately assessed in TMA cores of this size.

Luminal A-like (LumA) was defined as: ER+ and PR+ with nuclear grade 1/nuclear grade 2 with low Ki67. Luminal B-like (LumB) was defined as: ER+ and PR+ with nuclear grade 3/nuclear grade 2 with high Ki67. Her2-positive (Her2+) was defined as: Her2-staining 3+/amplified by SISH. Triple negative (TNBC) was defined as: ER-, PR- and Her2-.

### 3.6 Validation Cohort

To analyze the mRNA expression of type IV collagen genes (COL4A1, COL4A2, COL4A3, COL4A4, COL4A5 and COL4A6), a cohort from the Cancer Genome Atlas (TCGA) was obtained. The TCGA database contains mRNA sequence data and clinicopathological data from different cancer forms. Clinical data and fragments per kilo base of transcript per million mapped reads at upper quartile (FPKM-UQ) were obtained from the available primary breast cancer patients in the TCGA (*n* = 1,104) (The Cancer Genome Atlas database 2021). The FPKM-UQ data is generated by first aligning RNA-Seq reads with reference genome followed by normalization using the FPKM-UQ method (https://docs.gdc.cancer.gov/Data/Bioinformatics_Pipelines/Expression_mRNA_Pipeline/) The database was last updated 29th of October, 2021.

### 3.7 Statistics

The mean type IV collagen expression of the two cores from each tumor was calculated. If only one of the cores was available a single score was used. Low expression was defined as a mean value below 1.5; moderate expression as mean value between 1.6 and 2.0 and high expression as 2.1 or higher. Tissues with high expression were clear and easy to distinguish from those with a moderate to low expression. Thus, sections scored as having a low or moderate expression were more similar in appearance. Due to this observation, in the statistical analysis, low and moderate expression groups (mean value below 2.0) were combined and defined as low type IV collagen expression (*n* = 621). The high expression group was smaller in size (*n* = 93). Differences between type IV collagen expression and pathological parameters was calculated using Pearson´s chi-square test. Probability of survival was estimated using the Kaplan-Meier estimator and differences were analyzed with the log-rank test. The survival time used in the analyses was 10 years. Univariable and multivariable Cox regression analysis was used for survival analyses. The variables used in the multivariable analysis were clinically established and used prognostic factors such as age, tumor size, molecular subtype and adjuvant treatment.

High mRNA expression of COL4A1, COL4A2, COL4A3, COL4A4, COL4A5, and COL4A6 genes was defined as levels above the third percentile, and low expression as levels below. To compare the level of expressed genes to different clinicopathological parameters, independent T test and Pearson´s chi-square test was used. Probability of overall survival was estimated similarly as for the protein expression data. Clinical variables used in the multivariable analysis were age, TNM stage and radiotherapy treatment. SPSS statistics software for Windows, version 24 (IBM Corp., Armonk, N.Y., United States) was used for statistical analysis. In all tests a significance level of 5% was used.

## 4 Results

### 4.1 Cohort Characterization

**FIGURE 1:**
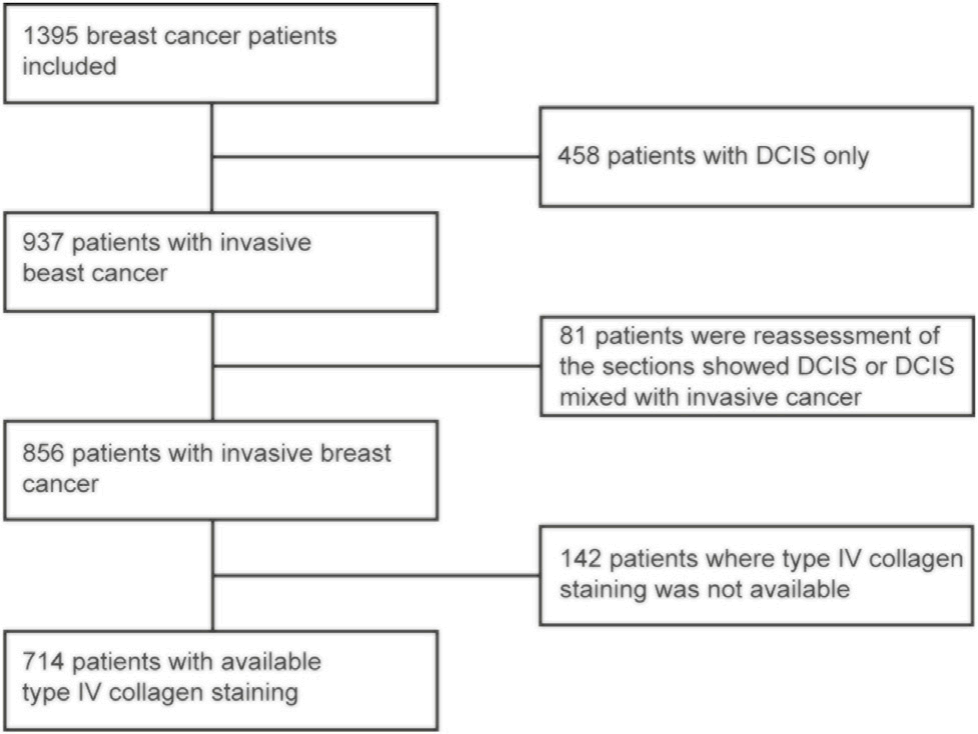
Flow chart for selection of the patients for the final study cohort.

Initially, 1,395 patients with clinically small breast cancers were included in the cohort. Out of these, 539 patients only had DCIS and were excluded from the present analysis. After this exclusion, 856 patients remained and out of these, type IV collagen staining was available for 714 patients (Figure 1). The median follow-up time was 11 years and 11 months, ranging from 2 months to 29 years and 7 months. No patients were lost to follow-up. The 5- and 10-year breast cancer specific survival (BCSS) was 96.2 and 92.8%, respectively. The mean age at breast cancer diagnosis was 60 years, ranging from 25 to 92 years, and most cancers (65.4%) were detected within the mammography screening program. In line with the inclusion criteria of clinically small cancers, the mean and median tumor sizes were 12.1 and 11.0 mm, respectively. Most cancers were ER and PR positive. Detailed patient and tumor characteristics are presented in Table 1.

**TABLE 1:** Patient characteristics of the cohort.

**Tumor size**	n	%
<20mm	638	89,4%
>21 mm	36	5,0%
Multifocal	40	5,6%
Grade		
1	54	7,6%
2	413	57,8%
3	173	24,2%
Data missing	74	10,4%
HER-2		
Positive	35	4,9%
Negative	610	85,4%
Data missing	69	9,7%
ER		
Positive	567	79,4%
Negative	63	8,8%
Data missing	84	11,8%
PR		
Positive	457	64,0%
Negative	177	24,8%
Data missing	80	11,2%
Ki-67		
High	311	43,6%
Low	303	42,4%
Missing	100	14,0%
Molecular subtype	N	%
LumA	261	36,6%
LumB	267	37,4%
TNBC	5 I	7,1%
HER2+	36	5,0%
Data missing	99	13,9%
Axillary status		
Metastasis	135	18,9%
No metastasis	579	81,1%
Surgical procedure		
Mastectomy	120	78,2%
BCS	588	82,4%
Biopsy	6	0,8%
Radical margins		
Yes	673	94,3%
No	4	0,6%
Doubtful	26	3,6%
Data missing	11	1,5%
Endocrine therapy		
Yes	233	32,6%
No	476	66,7%
Data missing	5	0,7%
Radiotherapy		
Yes	558	78,2%
No	156	21,8%
Chemotherapy		
Yes	87	12,2%
No	627	87,8%

### 4.2 Expression of Type IV Collagen

**FIGURE 2:**
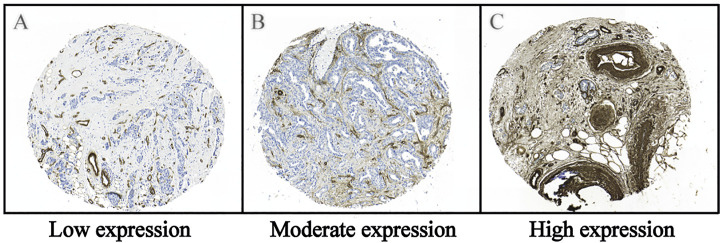
Representative images of the immunohistochemical staining of type IV collagen in the tumor stroma of breast cancer tissue. **(A)** Low expression, **(B)** Moderate expression and **(C)** High expression of type IV collagen in the tumor stroma. Immunohistochemical staining for type IV collagen was performed in the paraffinized TMA tissues using a rabbit anti-collagen IV (AB748, Millipore, Billerica, United States) antibody diluted 1:50, followed by a biotinylated secondary antibody diluted 1:200 and diaminobenzidine tetrahydrochloride (DAB) as a chromogen. For additional details see the Material and Methods section.

**FIGURE 3:**
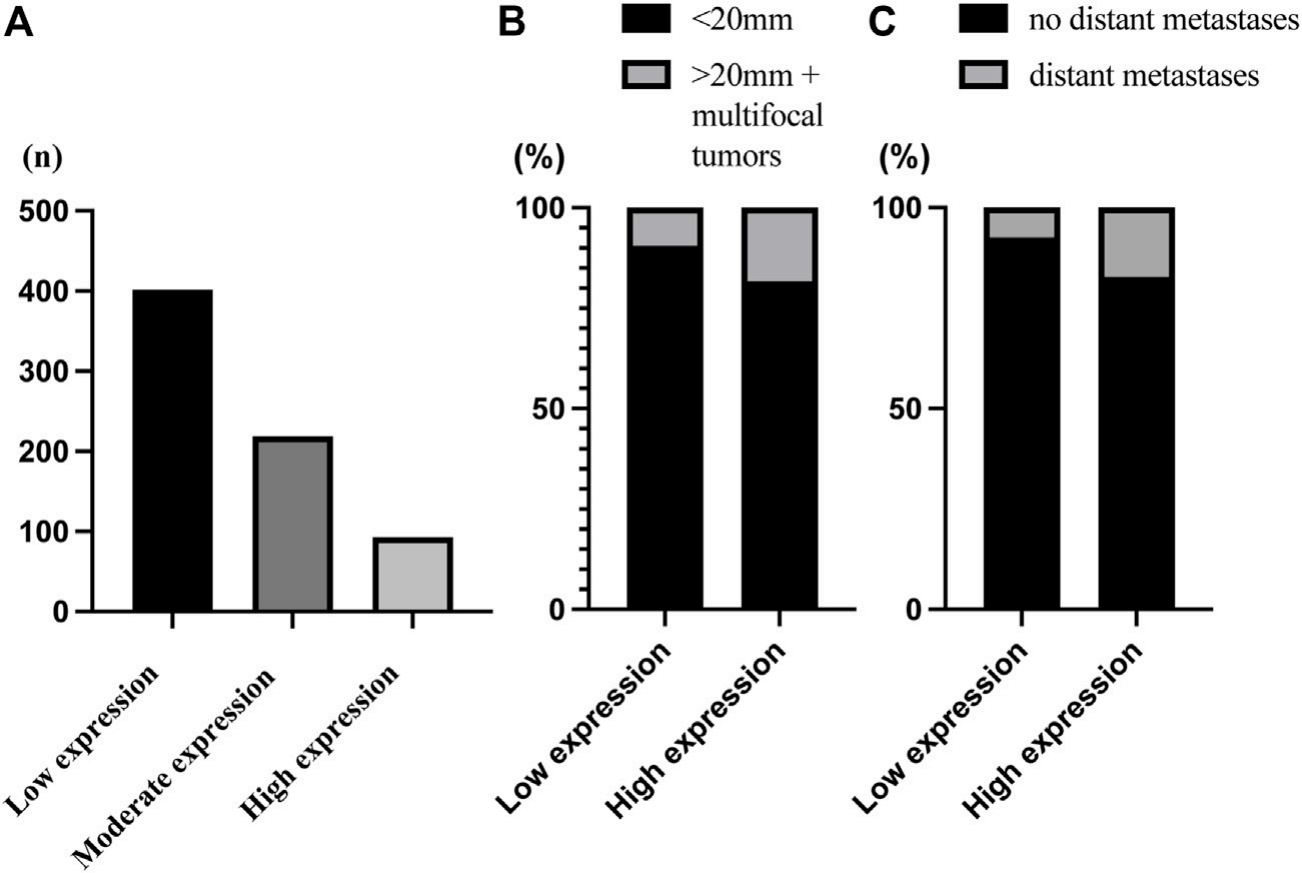
Bar chart of the distribution of type IV collagen expression. **(A)** The distribution of the type IV collagen scoring groups defined as low expression (*n* = 402), moderate expression (*n* = 219) and high expression (*n* = 93). **(B)** There was a significant correlation between tumor size and type IV collagen expression (*p* = 0.01). There was a higher proportion of small tumors (90.5%) in the low compared to the high type IV collagen expression group (81.7%), and a higher proportion of large tumors (>20 mm or multifocal tumors) (18.3%) in the high expression group compared with the low type IV expression group (9.5%). **(C)** There was a significant correlation between distant metastasis and type IV collagen expression (*p* = 0.002). There was a higher proportion of distant metastasis (17.2%) in the high compared to the low type IV collagen expression group (7.4%), and a higher proportion of no distant metastasis in the low (92.6%) compared to the high type IV collagen expression group (82,8%).

Type IV collagen in normal breast tissue was expressed both in the epithelial and vascular BMs and absent in the stroma (Supplementary Figure S1). In breast cancer tissue, type IV collagen was expressed in varying extent in the tumor stroma (Figure 2). Most breast cancers had a low or a moderate type IV collagen stromal expression score (Figure 3A). There was no correlation of type IV collagen expression to ER status (*p* = 0.27), PR status (*p* = 0.51), Her2 amplification (*p* = 0.8), Ki67 level (*p* = 0.48), nuclear grade (*p* = 0.85), molecular subtype (*p* = 0.15) or axillary status (*p* = 0.068). A significant correlation (*p* = 0.01) between tumor size and type IV collagen expression was observed. Tumors larger than 20 mm or multifocal cancers more often had a high type IV collagen expression (Figure 3B). There also was a significant correlation between age and type IV collagen expression (*p* = 0.02), with an older mean age (61 years) in the low expression groups compared with the moderate (59 years) and high expression groups (57 years).

### 4.3 Type IV Collagen Expression and Survival Analyses

During the first 10 years of follow up time, a total of 144 (20.2%) women died, of whom 47 (6.6%) died of breast cancer.

**FIGURE 4:**
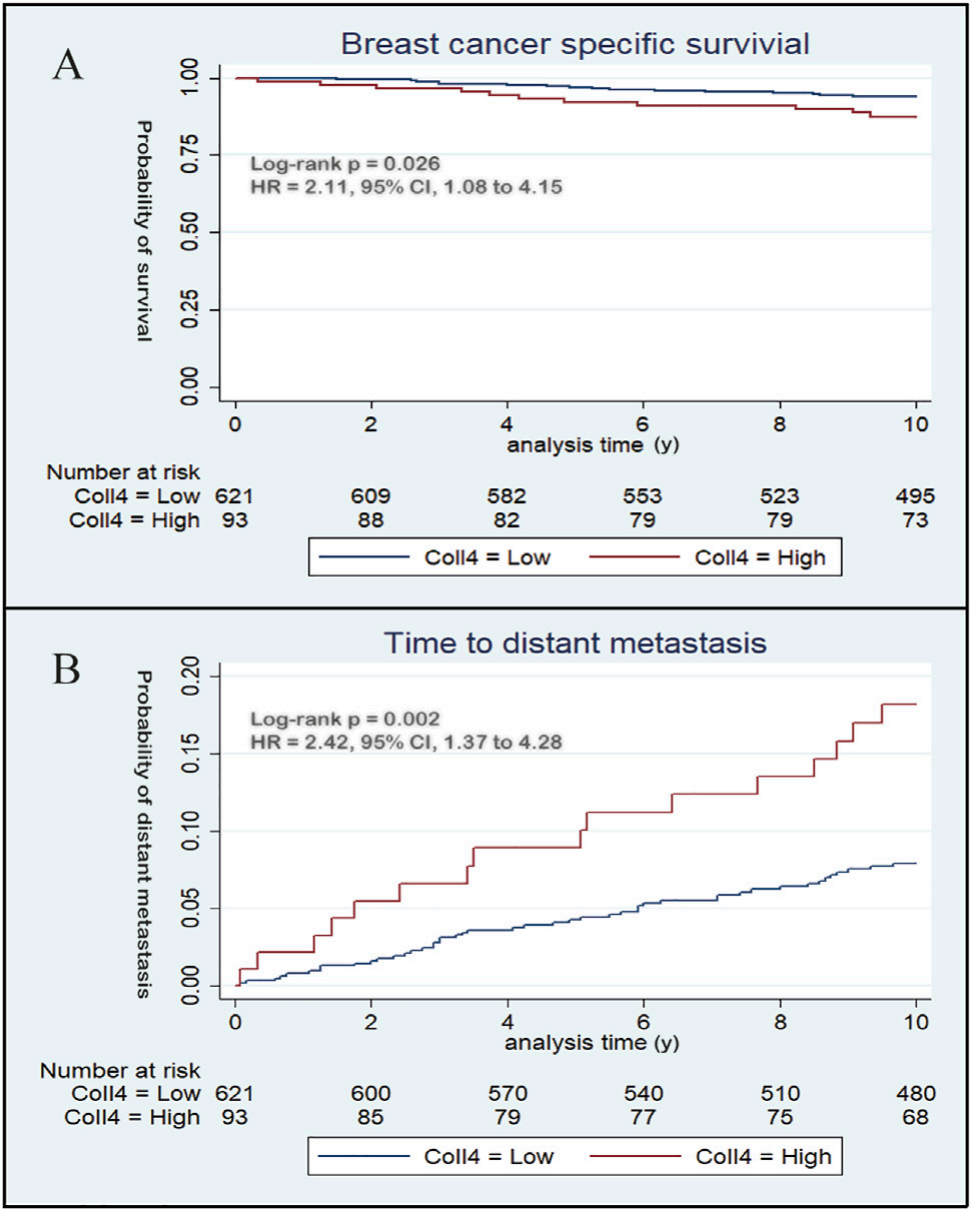
Kaplan-Meier curves demonstrate the prognostic potential of type IV collagen. **(A)** During the first 10 years of follow up, high expression of type IV collagen was associated with a poorer breast cancer specific survival compared to low expression of type IV collagen and **(B)** high expression of type IV collagen was positively associated with distant metastasis.

Kaplan-Meier analysis showed that high stromal type IV collagen expression in the primary tumor was significantly associated (*p* = 0.026) with a poorer breast cancer specific survival (BCSS) (Figure 4A). In univariable analysis, type IV collagen expression was significantly associated with BCSS (Supplementary Table S1), and when adjusted for tumor- and patient related prognostic factors, the association of type IV collagen expression to BCSS remained significant (*p* = 0.038) (Table 2). No statistically significant differences (*p* = 0.66) in overall survival were seen between patients with high and low type IV collagen expression.

**TABLE 2:** Multivariable Cox regression analysis of clinicopathological variables affecting breast cancer specific survival. High type IV collagen expression, tumor size larger than 20 mm or multifocal tumors, Her2+ and TNBC molecular subtypes, axillary metastasis and no radiotherapy significantly affects breast cancer specific survival in this cohort.

Characteristics	Hazard ratio	Unfavourable / Favourable	p-value	95% CI
Type IV collagen expression	2.08	High / low	0.038	1.04–4.17
Age (years)	1.37	>61 / <60	0.367	0.69–2.72
Size (mm)	2.20	>20+multifocal / <20	0.029	1.08–4.49
Molecular subtype	2.90	Her2+ and TNBC / LumA and LumB	0.002	1.48–5.63
Axillary status	5.7	Metastases / No metastases	0.000	2.41–13.48
Radiotherapy	2.14	No / Yes	0.050	1.00–4.58
Chemotherapy	1.9	No / Yes	0.149	0.80–4.50
Endocrine therapy	1.44	No / Yes	0.319	0.70–2.97

### 4.4 Type IV Collagen Expression and Distant Metastatic Disease

A total of 62 (8.7%) patients developed distant metastases during the first 10 years of follow up. Distant metastatic disease was significantly (*p* = 0.002) correlated to stromal type IV collagen expression in the primary tumor. In patients with high type IV collagen expression, 17.2% developed distant metastasis, whereas only 7.4% of patients with low stromal type IV collagen expression developed distant metastases (Figure 3C). Kaplan-Meier analysis showed that patients with high type IV collagen expression had a significantly higher frequency of distant metastases compared to those with low expression (*p* = 0.002) (Figure 4B). In the univariable analysis, type IV collagen expression was a significant (*p* = 0.002) risk factor for distant metastasis (Supplementary Table S2). When adjusted for clinically relevant tumor- and patient related prognostic factors in a multivariable analysis, a trend towards type IV collagen being an independent prognostic variable for distant metastasis was found, although the finding did not reach statistical significance (*p* = 0.076) (Table 3). During the whole follow up time only 19 patients (2.7%) developed lymph node recurrence and due to the low number of events no further analyses were made.

**TABLE 3:** Multivariable Cox regression analysis of clinicopathological variables affecting metastatic disease. Tumor size larger than 20 mm or multifocal tumors, Her2+ and TNBC molecular subtypes, axillary metastasis, and no radiotherapy received significantly affects distant metastasis in this cohort.

Characteristics	Hazard ratio	Unfavourable / Favourable	p-value	95% CI
Type IV collagen expression	1.77	High / Low	0.076	0.94–3.31
Age (years)	0.80	>61 / <60	0.472	0.43–1.48
Size (mm)	2.16	>20+multifocal / <20	0.019	1.13–4.10
Molecular subtype	2.48	Her2+ and TNBC / LumA and LumB	0.003	1.37–4,49
Axillary status	4.41	Metastases / No metastases	0.000	2.00–9.72
Radiotherapy	2.09	No / Yes	0.035	1.05–4.17
Chemotherapy	1.52	No / Yes	0.288	0,70–3.30
Endocrine therapy	1.76	No / Yes	0.094	0.91–3.41

### 4.5 Type IV Collagen Gene Expression in the Validation Cohort

The validation cohort consisted of 1,104 patients with primary breast cancer. In seven patients, mRNA expression levels were was also available from metastatic tissue. The median follow-up time was 28 months, and the mean age was 58 years. Detailed patient and tumor characteristics for this cohort are presented in Supplementary Table S3. The mRNA expression level of all six type IV collagen genes (COL4A1, COL4A2 COL4A3, COL4A4, COL4A5 and COL4A6) was higher in metastatic tissue compared to primary tumor tissue, although being significant only for COL4A3 (*p* = 0.013) and COLA4A4 (*p* = 0.005) genes (Supplementary Figure S2). In survival analyses high COL4A1 gene expression was associated with poorer overall survival (*p* = 0.041), and the difference remained significant even when adjusted for other prognostic factors (*p* = 0.011) (Supplementary Table S4).

## 5 Discussion

In this large population-based cohort of women with small breast cancers, high expression of type IV collagen in the tumor stroma was associated with a poorer breast cancer specific survival. There was no empirical evidence of correlation between expression of type IV collagen in the tumor stroma and other known prognostic biomarkers, except for tumor size. Larger tumors more often had a high expression score of type IV collagen, although due to the inclusion criteria even the larger tumors were relatively small in the present cohort. High expression of type IV collagen in the tumor stroma did not significantly correlate to axillary metastasis, but was highly correlated to distant metastatic disease. In the validation cohort, high levels of COL4A1 gene expression correlated significantly with poorer overall survival, a finding in line with the data on protein expression. Unfortunately, data regarding breast cancer specific survival was not available in this dataset. Most interestingly, the validation cohort included samples from metastatic breast cancer tissue and in these higher type IV collagen mRNA levels were found compared to primary breast cancer tissue.

At the early stages of cancer invasion, the loss of type IV collagen α-chains from the epithelial BM have been reported in several types of cancer (Ikeda et al., 2006; Baba et al., 2007). The BM becomes fragmented or even completely lost in the progression of cancer, and this was also seen in the present study (data not shown). Interestingly, type IV collagen deposits have been observed in the tumor stroma of several types of cancer (Amenta et al., 2000; Ioachim et al., 2002; Ohlund et al., 2009). In the present study, type IV collagen was expressed in the tumor stroma at varying levels, but in some of the breast cancers the expression was very intense and prominent. Survival analyses showed a poorer breast cancer specific survival in breast cancer patients with high stromal type IV collagen protein expression and a poorer overall survival in patients with high expression of type IV collagen mRNA. Previous research has shown that cancer cells modify the distribution of collagen in the stroma (Sugiyama et al., 2010; Kenny et al., 2017), and that type IV collagen interacts with cancer cells to promote migration (Favreau et al., 2014), stimulate proliferation, and inhibit apoptosis (Ohlund et al., 2013). Type IV collagen can also regulate angiogenic factors to promote tumor growth (Mammoto et al., 2013). Thus, this shift of type IV collagen localization, from the BM into the tumor stroma, would potentially contribute to a matrix more suited for tumor expansion. No correlations between type IV collagen expression and other known prognostic breast cancer biomarkers, except tumor size, could be seen in this study. Larger and multifocal tumors were shown to have a higher expression of type IV collagen, although there were very few large tumors in the cohort, and thus the statistical results regarding large tumors may be at risk of uncertainty.

Stromal type IV collagen expression in the primary tumor did not correlate to axillary metastasis. High stromal type IV collagen expression in the primary tumor however favored distant metastasis. In the multivariable analysis, type IV collagen expression no longer was a significant independent risk factor for distant metastatic disease, although the trend pointed towards a positive correlation between high stromal type IV collagen expression and a risk of developing distant metastasis.

It is possible that stromal type IV collagen can promote metastasis formation by supporting cancer cell survival and tumor progression, and high levels of type IV collagen in the metastases appear to be beneficial for metastatic growth. In line with this, higher levels of type IV collagen mRNA expression was found in metastatic compared to primary breast cancer tissue. In colorectal liver metastasis, an up-regulation of type IV collagen protein expression has been reported (Nystrom et al., 2011; Rolff et al., 2016), and type IV collagen was also shown to be highly expressed in tissues of both breast cancer liver and bone metastasis (Lindgren et al., 2021). It is well known that there often is a concordance in the relationship between pathological characteristics of the primary tumor and its distant metastasis (Kroigard et al., 2016). This indicates that high type IV collagen expression in the primary tumor reflects the expression of type IV collagen in the metastatic lesion. We have recently shown that higher levels of circulating type IV collagen can be seen in patients with metastatic breast cancer compared to patients with primary breast cancer, and high levels of circulating type IV collagen correlate to a poorer prognosis (Lindgren et al., 2021). The metastatic process involves remodeling of the ECM, followed by production and degradation of tumor stroma components, and the release of stromal substances into the circulation. Increased circulating levels of type IV collagen might therefore be related to high levels of type IV collagen in the metastatic tumor and in this study, it was also shown that type IV collagen mRNA was overexpressed in metastatic breast cancer tissue. In conclusion, high stromal type IV collagen expression in the primary tumor could be of importance in surveillance programs for breast cancer patients. Breast cancer patients with increased stromal type IV collagen expression should perhaps undergo a more intensive follow up program due to the high risk of developing distant metastatic disease.

## Data Availability

The raw data supporting the conclusions of this article will be made available by the authors, without undue reservation.
