# Supplementary material for: Prognostic Value of Stromal Type IV Collagen Expression in Small Invasive Breast Cancers
Source: Front Mol Biosci. 2022 May 25;9:904526. doi: 10.3389/fmolb.2022.904526 (PMC9174894; doi:10.3389/fmolb.2022.904526)
Supplement: Supplementary file 1 [file DataSheet2.PDF]

Univariable Cox-analyses of risk factors for distant metastases

Characteristics	Hazard ratio	Unfavourable/favourable	p-value	95% CI
**Type IV collagen expression**	2.42	High / Low	0.002	1.37-4.28
**Age (years)**	0.57	>61/ ≤60	0.036	0.34-0.96
**Size (mm)**	3.90	>20+multifocal/ ≤20	0.000	2.25-6.75
**Molecular subtype**	3.72	Her2+ and TNBC/ LumA and LumB	0.000	2.16-6.41
**Axillary status**	3.11	Metastases/ No metastases	0.000	1.87-5.17
**Radiotherapy**	1.35	No/ Yes	0.314	0.75-2.41
**Chemotherapy**	0.48	No/ Yes	0.014	0.26-0.86
**Endocrine therapy**	0.48	No/ Yes	0.84	0.50-1.46
