# Supplementary material for: Prognostic Value of Stromal Type IV Collagen Expression in Small Invasive Breast Cancers
Source: Front Mol Biosci. 2022 May 25;9:904526. doi: 10.3389/fmolb.2022.904526 (PMC9174894; doi:10.3389/fmolb.2022.904526)
Supplement: Supplementary file 2 [file DataSheet4.PDF]

**Multivariable Cox-analyses of variables related to ovarall survival in the gene expression dataset**

| Characteristics | Hazard ratio | Unfavourable/ Favourable | p-value | 95% CI |
|---|---|---|---|---|
| **COLA4A1 mRNA level** | 2.53 | High/ Low | 0.011 | 1.24-5.17 |
| **Age (years)** | 2.67 | >58/ ≤58 | 0.013 | 1.23-5.78 |
| **Size** | 3.10 | T2-4/ T0 | 0.085 | 0.80-11.79 |
| **Radiotherapy** | 1.73 | No/ Yes | 0.143 | 0.83-3.60 |
| **Nodestatus** | 1.03 | N1-3/ N0 | 0.948 | 0.43-2.46 |
| **Distant metastasis** | 7.41 | Yes/ No | 0.000 | 0.80-11.79 |
