# Supplementary figures and images for: Prognostic Value of Stromal Type IV Collagen Expression in Small Invasive Breast Cancers
Source: Front Mol Biosci. 2022 May 25;9:904526. doi: 10.3389/fmolb.2022.904526 (PMC9174894; doi:10.3389/fmolb.2022.904526)

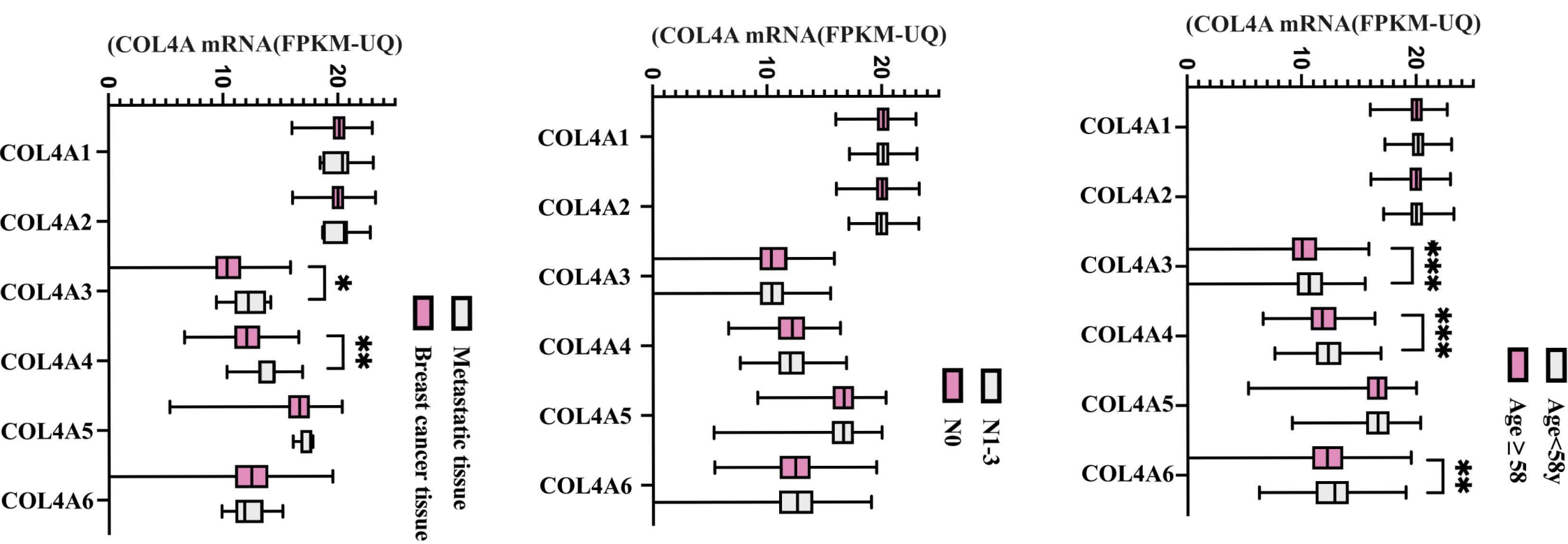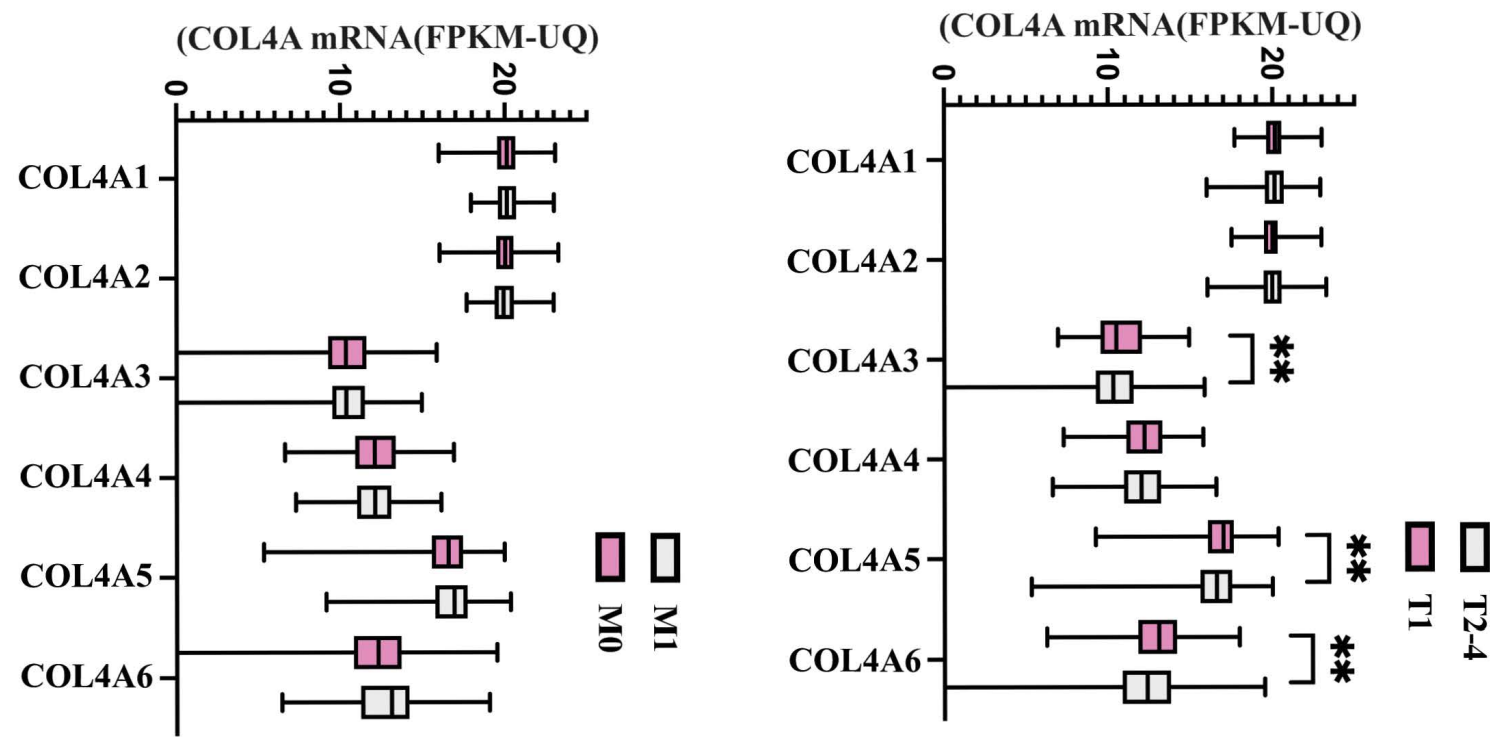

Supplement: Supplementary file 3 [file DataSheet6.PDF]

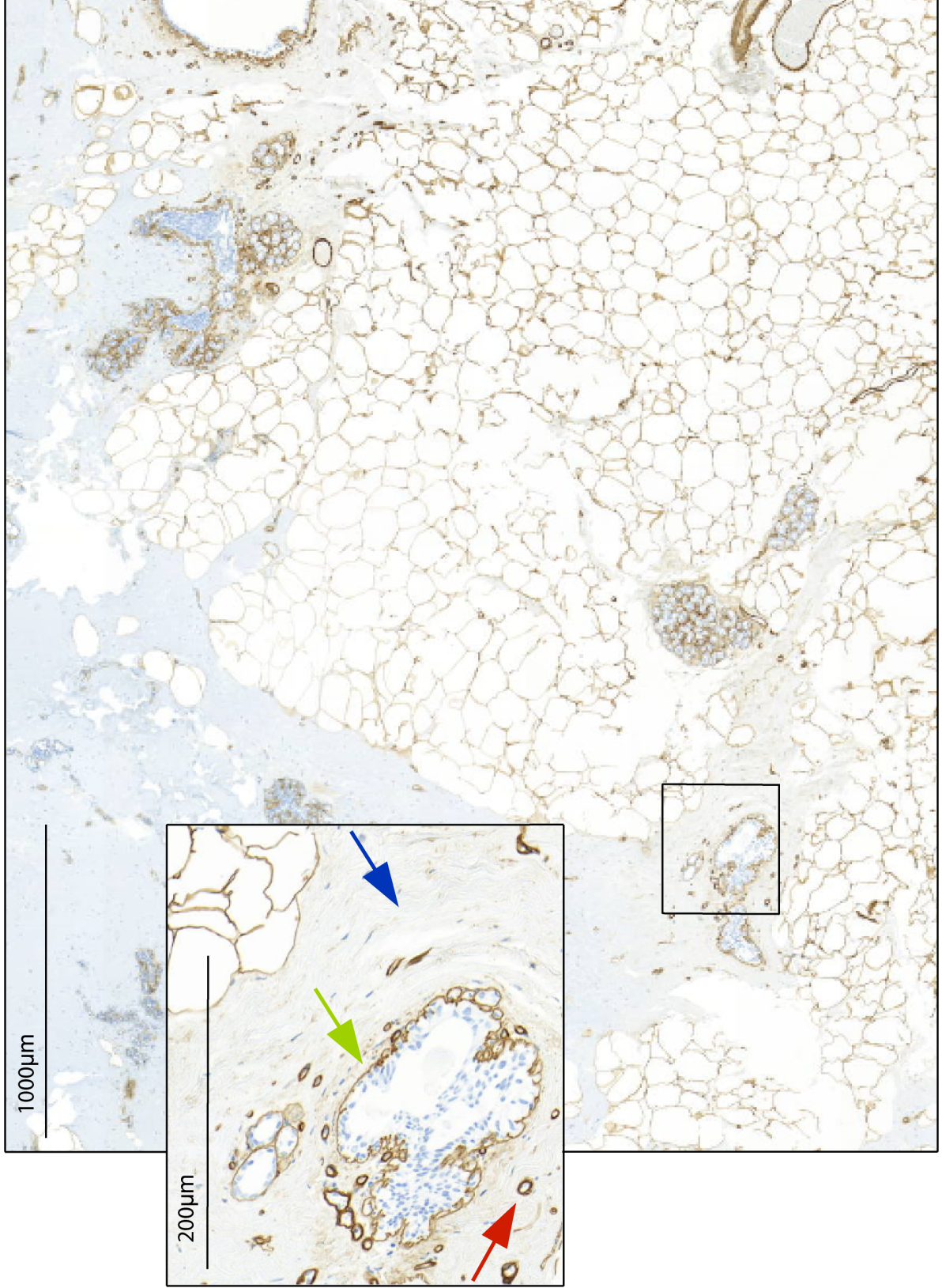

Supplement: Supplementary file 6 [file DataSheet5.PDF]
