# Supplementary material for: Prognostic Value of Stromal Type IV Collagen Expression in Small Invasive Breast Cancers
Source: Front Mol Biosci. 2022 May 25;9:904526. doi: 10.3389/fmolb.2022.904526 (PMC9174894; doi:10.3389/fmolb.2022.904526)
Supplement: Supplementary file 4 [file DataSheet3.PDF]

Patient characteristics in the gene expression dataset (n=1104)

T category	n	%
T1	182	*17,0%*
T2	163	*56,4%*
T3	218	*22,5%*
T4	20	*1,8%*
Missing	31	*2,8%*
**N category**		
N0	518	*46,9%*
N1	367	*33,2%*
N2	120	*10,9%*
N3	78	*7,1%*
Missing	21	*1,9%*
**M category**		
M0	911	*82,5%*
M1	22	*2,0%*
Missing	933	*15,5%*
**Molecular subtype**		
Luminal A	18	*1,6%*
Luminal B	24	*2,2%*
Her2	10	*0,9%*
Basal cell like	15	*1,4%*
Normal	1	*0,1%*
Unknown	4	*0,4%*
Missing	1032	*93,5%*
**Radical margin**		
Positive	78	*7,1%*
Negative	923	*83,6%*
Close	33	*3,0%*
Missing	70	*6,3%*
**Radiotherapy**		
Yes	431	*39,0%*
No	549	*49,7%*
Missing	124	*11,2%*
**Distant metastasis present**		
Yes	13	*1,2%*
No	385	*34,9%*
Missing	706	*69,9%*
