# Supplementary material for: Prognostic Value of Stromal Type IV Collagen Expression in Small Invasive Breast Cancers
Source: Front Mol Biosci. 2022 May 25;9:904526. doi: 10.3389/fmolb.2022.904526 (PMC9174894; doi:10.3389/fmolb.2022.904526)
Supplement: Supplementary file 5 [file DataSheet1.PDF]

Univariable Cox-analyses of risk factors for BCSS

Characteristics	Hazard ratio	Unfavourable/favourable	p-value	95% CI
**Type IV collagen expression**	2.11	High / Low	0.030	1.08-4.15
**Age (years)**	0.92	>61/ ≤60	0.784	0.52-1.64
**Size (mm)**	4.30	>20+multifocal/ ≤20	0.000	2.33-7.94
**Molecular subtype**	4.06	Her2+ and TNBC/ LumA and LumB	0.000	2.22-7.42
**Axillary status**	3.99	Metastases/ No metastases	0.000	2.25-7.08
**Radiotherapy**	1.63	No/ Yes	0.133	0.86-3.10
**Chemotherapy**	0.53	No/ Yes	0.077	0.27-1.07
**Endocrine therapy**	0.66	No/ Yes	0.163	0.37-1.18
